# Characterization of the complete mitochondrial genome of *Congrogadus subducens* (Richardson, 1843)

**DOI:** 10.1080/23802359.2022.2026830

**Published:** 2022-01-24

**Authors:** Jin Gao, Hongji Ke, Yongbo Wang, Wei Tan, Chuan Lin

**Affiliations:** a Ministry of Education, Key Laboratory of Utilization and Conservation for Tropical Marine Bioresources (Hainan Tropical Ocean University), Sanya, China; b Hainan Provincial Key Laboratory of Tropical Maricultural Technologies, Hainan Academy of Ocean and Fisheries Sciences, Haikou, China; c Department of Animal Cultivation, Hainan Agriculture School, Haikou, China

**Keywords:** *Congrogadus subducens*, mitochondrial genome, phylogenetic analysis

## Abstract

The complete mitochondrial genome of *Congrogadus subducens* is first presented in this study. The whole mitogenome is a closed circular molecule of 16,881 bp in size, including 13 protein-coding, 22 transfer RNA, 2 ribosomal RNA genes and a non-coding control region. The overall base composition of the mitochondrial DNA is 30.2% for A, 28.6% for T, 26.4% for C and 14.8% for G. The phylogenetic analysis conducted using 18 protein-coding genes showed that *C. subducens* was most closely related to the Pseudochromidae. This work will be useful for further research on species identification and evolutionary relationships within related species.

Carpet Eel Blenny, *Congrogadus subducens* Richardson 1843, belongs to the family Pseudochromidae. It is a species distributed in Indian Ocean, Okinoshima Island, Western Australian and Queensland (Winterbottom et al. 1984). The body of *C. subducens* with deep anal fin is very long, slender and eel-like. This species inhabits in coastal reefs and estuaries, and has a fancy for hiding itself amongst rocks, algae and coral rubble (Randall et al. 1990). Here, we described the characterization of the complete mitochondrial genome of *C. subducens* and explored the taxonomy and phylogeny among Perciformes species. Meanwhile, the complete mitogenome sequence also provided valuable genetic information for identifying and understanding diversity and evolution of the species more correctly.

The specimen of *C. subducens* was collected from Qionghai Coast (geographic location: 19°43′24ʺN, 110°77′32ʺE) of Hainan Province, the South China Sea. The tissues were preserved in 95% ethanol and deposited in Hainan Academy of Ocean and Fisheries Sciences (Jin Gao, gaojin427@126.com) with voucher number 20200718YP01. Total genomic DNA was extracted from the back muscle by using DNeasy Blood & Tissue Kit (Qiagen, Valencia, CA, USA). Sequencing libraries were constructed with the NexteraXT DNA Library Preparation Kit and sequenced with Illumina Novaseq platform (Total Genomics Solution Limited, SZHT). All sequenced fragments were assembled to create the complete mitogenome using the GetOrganelle v1.6.2e (Jin et al. 2020). Protein-coding genes (PCGs) and tRNA genes were identified using BLAST search in NCBI and the tRNAscan-SE search server (Schattner et al. 2005), respectively. The complete mitogenome was annotated and verified with the software of MITOS (Bernt et al. 2013).

The complete mitogenome of *C. subducens* was 16,881 bp in length (GenBank accession number: MW854239), containing 13 PCGs (*nad1*, *nad2*, *cox1*, *cox2*, *atp8*, *atp6*, *cox3*, *nad3*, *nad4l*, *nad4*, *nad5*, *nad6*, *cob*), 22 tRNA genes (*trnF-PHE*, *trnV-VAL*, *trnL2-LEU2*, *trnI-ILE*, *trnQ-GLN*, *trnM-MET*, *trnW-TRP*, *trnA-ALA*, *trnN-ASN*, *trnC-CYS*, *trnY-TYR*, *trnS2-SER2*, *trnD-ASP*, *trnK-LYS*, *trnG-GLY*, *trnR-ARG*, *trnH-HIS*, *trnS1-SER1*, *trnL1-LEU1*, *trnE-GLU*, *trnT-THR*, *trnP-PRO*), 2 rRNA genes (12S and 16S) and one non-coding control region. The overall nucleotide composition is 30.2% for A, 28.6% for T, 26.4% for C and 14.8% for G, with AT bias of 58.8%.

Mitochondrial genes are also used for species identification and inferring phylogenetic relationship within tree families (Frezal and Leblois 2008; Kochzius et al. 2010). Based on the 13 PCGs of *C. subducens* and other 17 related species, a phylogenetic analysis was performed using maximum-likelihood method of MEGA7.0 (Kumar et al. 2016) with 1000 bootstrap replicates, a Scophthalmidae species (*Scophthalmus maximus*) was selected as the outgroup. The phylogeography analysis presented here strongly supported *C. subducens* was closely related to *Labracinus cyclophthalmus* and *Pictichromis paccagnellae* with a bootstrap probability of 97% (Figure 1). In conclusion, our results might also be useful for guiding future research on identification and evolutionary relationships in Blenniiformes.

**Figure 1.**
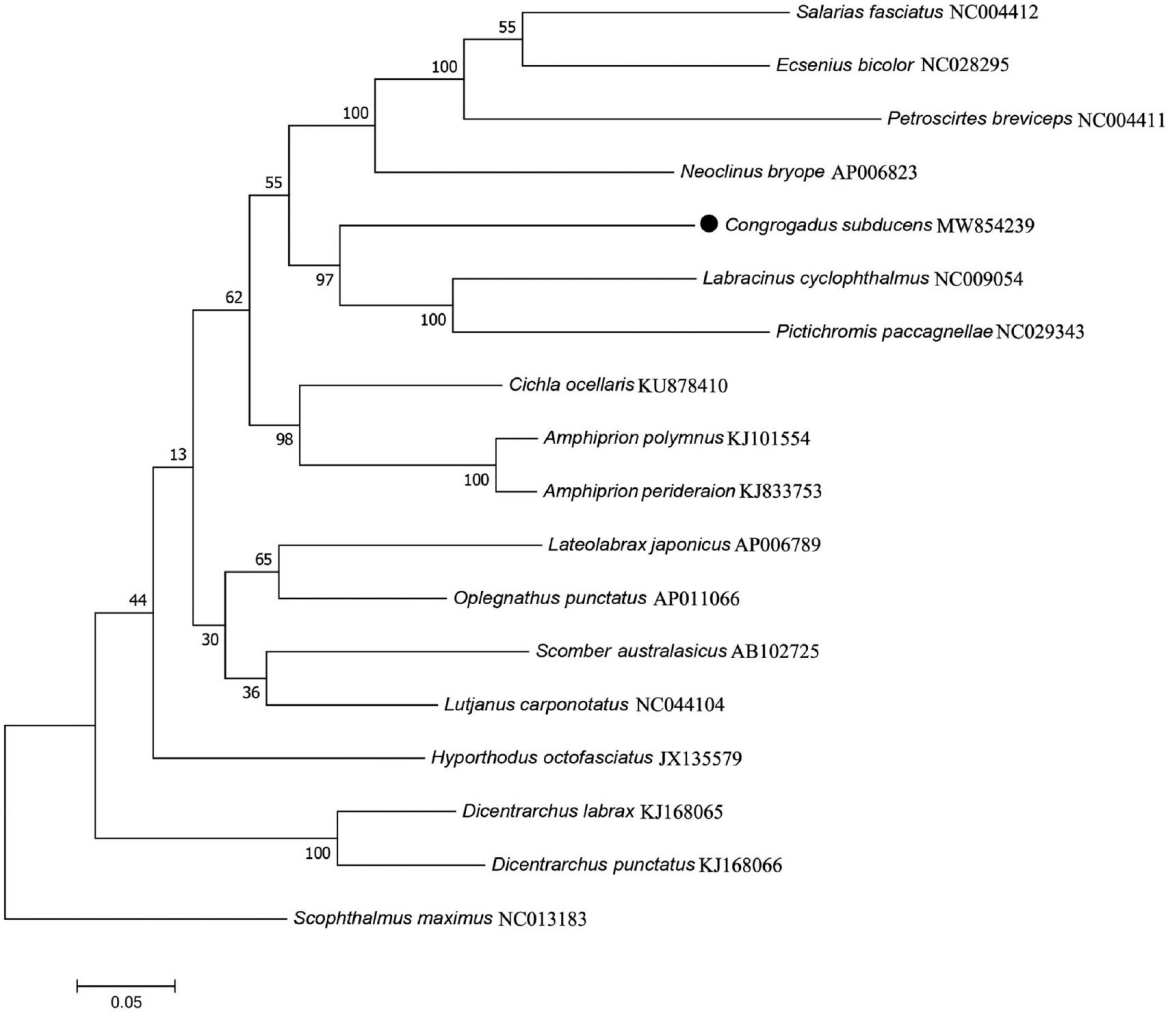
Maximum likelihood tree of 18 marine fishes was constructed based on 13 mitochondrial PCGs sequences and *Scophthalmus maximus* as outgroup. The numbers at the nodes are bootstrap probability based on 1000 replications. GenBank accession numbers of each sequence were listed in the tree with their corresponding species names.

## Ethical approval

Experiments were performed in accordance with the recommendations of the Ethics Committee of Hainan Academy of Ocean and Fisheries Sciences. These policies were enacted according to the Chinese Association for the Laboratory Animal Sciences and the Institutional Animal Care and Use Committee (IACUC) protocols.

## Data Availability

The genome sequence data that support the findings of this study are openly available in GenBank of NCBI at (https://www.ncbi.nlm.nih.gov/) under the accession no. MW854239. The associated BioProject, SRA, and Bio-Sample numbers are PRJNA748825, SRR15212447, and SAMN20345215 respectively.
